# The effects of social vs. personal power on universal dimensions of social perception

**DOI:** 10.3389/fpsyg.2022.1050287

**Published:** 2023-01-04

**Authors:** Linda Lai

**Affiliations:** Department of Leadership and Organizational Studies, BI Norwegian Business School, Oslo, Norway

**Keywords:** power, stereotyping, warmth, competence, personal power, social power, poor, rich

## Abstract

The present study expands previous research on the effects of power on stereotyping by investigating the impact of two types of power (social power and personal power) on two universal dimensions of social perception; warmth and competence. Results from an experiment (*N* = 377) in which participants were randomly assigned to provide their impression of either (1) poor people or (2) rich people, suggest that the two types of power produce different effects on perceptions of warmth and competence. Personal power increased stereotype consistent perceptions of warmth whereas social power increased stereotype consistent perceptions of competence as well as agency, which was identified as a separate dimension. The pattern of results is discussed in view of previous work on power effects and stereotyping, and potential explanations and suggestions for future research are outlined.

## Introduction

Stereotyping has been a central domain of inquiry in social psychology for decades, and a number of studies suggest that social power can affect the tendency to stereotype others. Fiske (1993: 621) proposed that stereotyping and power are closely interrelated and mutually reinforcing, and that powerful people stereotype more than the powerless in order to exert control and maintain and justify status quo. While some studies have found support for the proposition that power tends to increase stereotyping (e.g., Fiske and Dépret, 1996; Goodwin et al., 2000; Schmid and Amodio, 2017), other studies have shown that power can reduce stereotyping (e.g., Overbeck and Park, 2001; Chen et al., 2004; Mast et al., 2009). However, an increasing number of studies demonstrate that the effects of power can be moderated by individual as well as situational factors, including, for example, organizational culture (Overbeck and Park, 2006), the type of domain (Vescio et al., 2003, 2005), power legitimacy (Rodriguez-Bailón et al., 2000), and stereotype accessibility (Guinote and Phillips, 2010).

However, Lammers et al. (2009) argue that some of the inconsistencies in findings pertaining to positive vs. negative effects of power can be attributed to different conceptualizations of power. According to Lammers et al. (2009), some scholars have conceptualized power as social power, i.e., the ability to exercise influence over others, whereas other scholars have (implicitly) conceptualized power as personal power, i.e., personal agency, autonomy, and the freedom to resist influence from others (cf. Van Dijke and Poppe, 2006). A related distinction is presented by Scholl et al. (2020), who argue that the effects of power depend on how powerholders conceptualize their power with respect to opportunity vs. responsibility. When power is conceptualized as opportunity, powerholders experience freedom and independence. When power is conceptualized as responsibly, in contrast, powerholders experience higher commitment to goals and to taking care of others (Scholl et al., 2020).

Lammers et al. (2009) reasoned that since personal power is associated with *independence* from others, personal power should lead to increased stereotyping compared to social power, which is associated with *interdependence* and hence the need for interaction and responsibility, which should lead to reduced stereotyping. A similar line of reasoning was presented by Fiske (1993), who proposed that when people are interdependent, they pay more attention to stereotype-inconsistent information and hence stereotype less. Accordingly, Lammers et al. (2009: 1544) argued that when the distinction between independence and interdependence is relevant, the effects of social and personal power can differ, and that compared to social power, personal power should lead to increased stereotyping. However, Fiske’s (1993) proposition that power will increase stereotyping due to the need for control and maintaining the status quo, also seems particularly relevant for those who receive their power through interpersonal relations (i.e., social power). Hence, based on this line of reasoning, social power should lead to increased rather than decreased stereotyping compared to personal power.

However, Lammers et al. (2009) reported empirical support for their proposition based on a survey study as well as an experimental study. A study by Mayiwar and Lai (2019) partially replicated the results from the experiment, yet effect sizes were considerably smaller and neither social nor personal power differed significantly from the control condition. Hence, future research is needed in order to gain better insight into the relative effects of social vs. personal power on stereotyping.

Lammers et al. (2009) and Mayiwar and Lai (2019) measured stereotyping by means of an unidimensional measure of the degree to which a woman depicted in a short story was perceived as stereotypically female. However, a large body of research suggests that social perception and stereotyping tend to be structured according to two universal and distinct dimensions: warmth and competence (e.g., Fiske et al., 2002, 2007; Cuddy et al., 2008). Fiske et al. (2002) propose that warmth and competence reflect different needs in social interaction; the need to understand others’ intent (warmth) vs. the need to know others’ effectiveness in pursuing their goals (competence). Hence, the distinction between warmth and competence is similar to the distinction between communion and agency, yet not entirely overlapping (Cuddy et al., 2008).

So far, few if any studies have investigated the effects of social vs. personal power on stereotyping that involves warmth and competence. Accordingly, the main purpose of the present study is to contribute to filling this gap in the literature by investigating whether social power and personal power produce different effects on perceptions of warmth and competence.

For the purpose of the present research, two social groups that tend to be viewed as outgroups and ambivalently stereotyped, yet in the opposite direction, were chosen for inquiry: rich people and poor people, respectively. Poor people tend to be perceived as higher in warmth than competence, whereas rich people tend to be perceived as higher in competence than warmth (e.g., Bye et al., 2014; Lindqvist et al., 2017; Bryksina et al., 2021).

Based on the line of reasoning by Lammers et al. (2009), it may be expected that social power will produce less stereotypical perceptions of warmth and competence within and between groups. Yet, it also seems plausible to expect that social and personal power will differ with respect to the effects on perceptions of warmth and competence due to the inherent nature of the two dimensions. Fiske et al. (2007) argued that while warmth represents a moral-social trait that is *other-profitable,* i.e., affects other people, competence represents a *self-profitable* trait, i.e., affects the possessor and his/her odds of goal-achievement.

Based on the line of reasoning above, the following exploratory hypothesis was formulated for empirical inquiry in the present study:

*Hypothesis*: Social and personal power will have different effects on stereotyping of warmth and competence.

## Materials and methods

### Design and procedure

An online experiment was conducted to test the hypotheses. Participants who provided their informed consent took part online on a voluntary basis and rated their own social and personal power before being randomly assigned to rate either poor people or rich people.

In the vignette, participants were instructed to “think about people that you have either read about, heard about, or know yourself that you have reason to believe (struggle very hard financially/are very well off financially) and that are likely to be viewed as (poor/rich),” and to provide their personal impression of the given group.

### Participants

Participants were recruited on Facebook and LinkedIn. A sample of no less than 300 (150 in each experimental group) was aimed for in order to reach satisfactory statistical power (>0.80) to detect small effect sizes in multiple regression analyses with up to five predictors (cf. Cohen, 1988). 377 participants completed the experiment. Power analysis using the “Post-hoc Statistical Power Calculator for Multiple Regression” indicates that observed statistical power reaches 0.947 (*p* ≤ 0.05) based on the current sample size. 74.3% of participants were female. Age was measured in 10-year intervals, with a median of approximately 50 years old. 34% held a bachelor’s degree, while 52% held a master’s degree or PhD.

### Measures

*Social power* was measured with eight items drawn from the Sense of Power scale (Anderson et al., 2012), which has been used in previous research to differentiate between social and personal power (e.g., Leach et al., 2017). All items were retained after principal component analyses and coefficient alpha tests (α = 0.878), e.g., “I can easily get other people to do what I want,” “My ideas and opinions are rarely ignored,” and “If I want to, I get to make the decisions.”

*Personal power* measures were based on a total of seven items that were inspired by the priming task in Lammers et al. (2009) as well as the personal control scale by Cichocka et al. (2018). Following principal component analyses and coefficient alpha tests, two items that loaded on a separate factor were excluded, while five items were retained (α = 0.821), e.g., “I feel I have great control over what happens in my life,” “I feel free to do what I wish,” and “I feel that no one can force me to do anything against my will.”

*Warmth* was initially measured with five items (on a scale from 1 to 7), of which four (friendly, warm, good-natured, and sincere) were drawn from Cuddy et al. (2008) and one (predatory) was adapted from Lindqvist et al. (2017). The last item was removed based on principal component analysis and scale reliability tests, while all items drawn from Cuddy et al. (2008) were retained (α = 0.909).

*Competence* was initially measured with four items (on a scale from 1 to 7) drawn from Cuddy et al. (2008; competent, confident, capable, and skillful) and four items adapted from Lindqvist et al. (2017) and Bye et al. (2014): hard-working, keeping personal finances in order, taking care of ones’ health, and prioritizing education. Principal component analysis revealed two separate dimensions. Three items drawn from Cuddy et al. (2008), i.e., competent, capable, and skillful, loaded on one dimension and demonstrated satisfactory scale reliability (α = 0.875) while one item (confident) loaded on several factors and was removed.

The four items drawn from Lindqvist et al. (2017) and Bye et al. (2014) loaded on a separate factor (α = 0.851), which may be conceptualized as the level of *agency* attributed to the target social group. These results correspond to previous research that indicates that competence and agency represent distinct dimensions in social perceptions (e.g., Carrier et al., 2014). Cuddy et al. (2008) argue that although agency is closely associated with competence, agency not fully captures competence since agency is related to taking effective action, i.e., that competence may provide the potential for action but does not necessarily produce actual action. Hence, agency tends to be conceptualized as a subdimension of competence (Abele et al., 2008). Both dimensions were therefore retained for further analysis.

All independent and dependent variables were measured on a scale from 1 (strongly disagree) to 7 (strongly agree).

*Demographic control-variables* included gender, age (measured in 10-year intervals), education level, income level, and self-estimated financial status (from poor to very good).

## Results

### Initial analyses

Poor people were rated close to scale midpoint on competence (M = 4.007, SD = 0.913) and agency (M = 4.010, SD = 0.986). The scores on warmth were significantly higher than the scores on competence [M_Warmth_ = 4.813, SD = 0.882, t(201) = 12.753, *p* < 0.001, *d* = 0.987] and agency [*t*(201) = 11.196, *p* < 0.001, *d* = 0.788].

Rich people, in contrast, were rated close to scale midpoint on warmth (M = 3.900, SD = 0.791) and significantly higher on competence [M_Comp_ = 4.655, SD = 1.001, *t*(174) = 10.045, *p* < 0.001, *d* = 0.759] and agency [M_Agency_ = 5.179, SD = 0.823, *t*(174) = 17.626, *p* < 0.001, *d* = 1.332] compared to warmth. The scores on competence and agency were also significantly different [*t*(174) = 7.712, *p* < 0.001, *d* = 0.583].

When comparing rich people and poor people, ratings were significantly different across all dependent variables. Poor people were rated as significantly warmer [t(375) = 10.593, Mdiff = 0.913, *p* < 0.001, d = 1.085] than rich people, yet significantly lower on competence [t(375) = −6.536, Mdiff = −0.649, *p* < 0.001, d = −0.679] and agency [*t*(375) = −12.541, Mdiff = −1.169, *p* < 0.001, d = −1.279]. Hence, the results reveal that the rich as well as the poor were ambivalently stereotyped, yet in a reversed pattern, as expected.

### Hypothesis testing

The moderators of interest (social and personal power) were assessed as continuous variables. Hence, a set of regression analyses was performed after coding experimental condition as-1 (poor) or 1 (rich), centering social power and personal power scores, and calculating the interaction terms.

**Figure 1:**
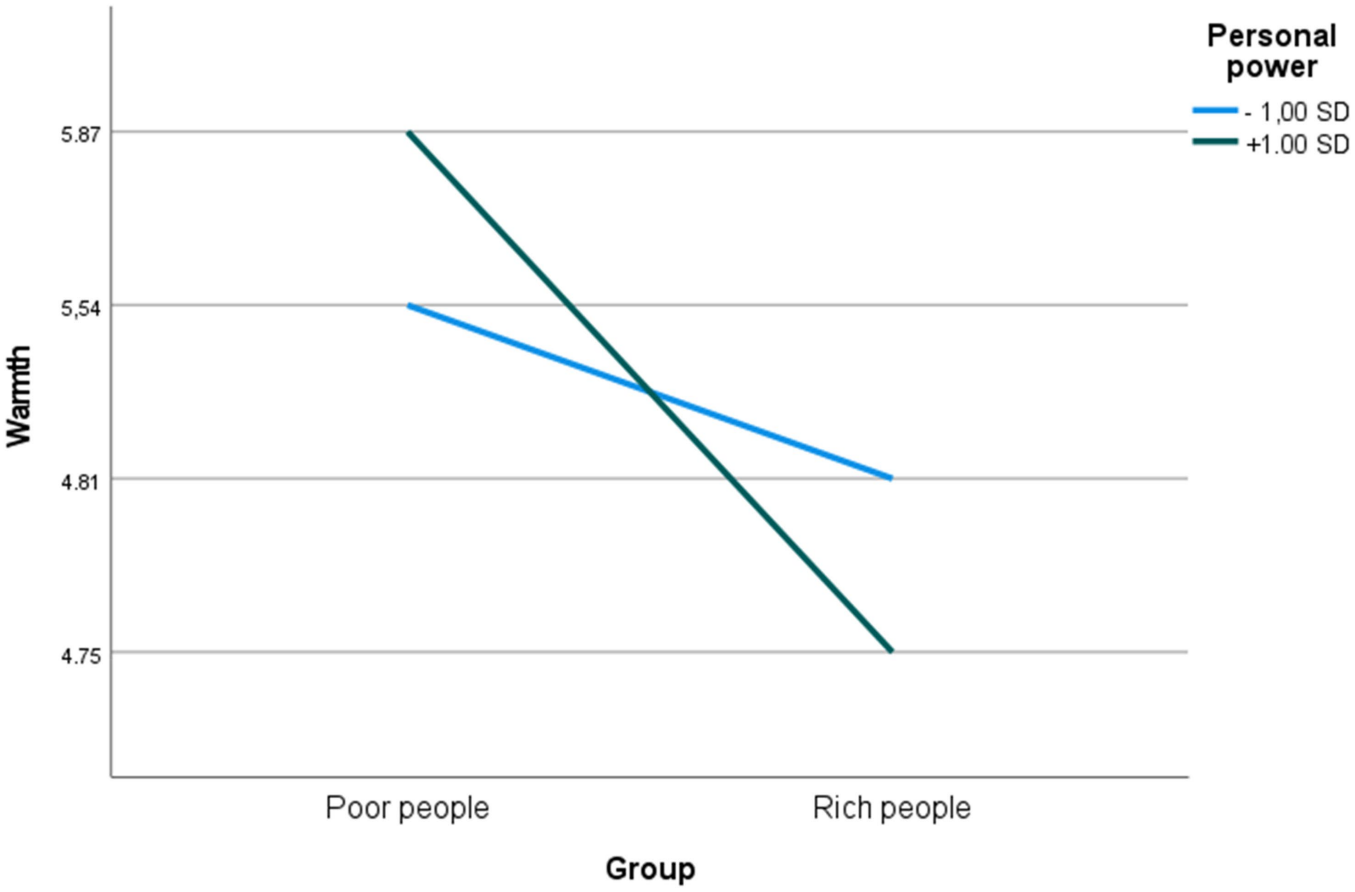
Predicted perceptions of warmth at high and low levels of personal power.

Results (see Table 1) reveal that after controlling for demographic variables, personal power moderated perceptions of warmth (β = −0.127, t = −2.669, *p* = 0.008), but not perceptions of competence or agency. Simple slope analyses (Aiken and West, 1991) showed that the simple slope was significant for high personal power (one standard deviation above the mean; ß = −0.542, t = −8.530, *p* < 0.001) as well as for low personal power (one standard deviation below the mean; ß = −0.436, t = −6.826, *p* < 0.001), indicating that high as well as low personal power increased the difference between poor people and rich people in ratings of warmth, i.e., the degree to which rich people were perceived as less warm compared to poor people. The two slopes were significantly different (t = 2.475, df = 750, *p* = 0.014), indicating that differences in perceptions of warmth were larger at high compared to low personal power (see Figure 1).

**Table 1:** Regression results—direct and moderated effects on perceptions of warmth, competence, and agency.

	Warmth (M = 4.39/SD = 0.96)				Competence (M = 4.31/SD = 1.01)			Agency (M = 4.55/SD = 1.08)		
	M(SD)	1	2	(95% CI)	1	2	(95% CI)	1	2	(95% CI)
Group (−1 = poor, 1 = rich)		−0.476^**^	−0.486^***^	(−0.550; −0.382)	0.325^***^	0.321^***^	(0.228; 0.419)	0.544^***^	0.541^***^	(0.494; 0.679)
Social power	4.86 (0.75)	0.113^*^	0.127^*^	(0.039; 0.286)	0.073	0.068	(−0.049; 0.231)	0.050	0.058	(−0.053; 0.220)
Personal power	5.56 (0.85)	0.031	0.031	(−0.072; 0.141)	0.059	0.056	(−0.055; 0.188)	0.072	0.061	(−0.040; 0.196)
Social power*group		0.091	0.096^*^	(0.003; 0.242)	0.251^***^	0.252^***^	(0.203; 0.474)	0.119^**^	0.119^**^	(0.041; 0.305)
Personal power*group		−0.135^**^	−0.127^**^	(−0.248; −0.038)	−0.064	−0.062	(−0.192; 0.046)	0.016	0.025	(−0.085; 0.147)
Gender(1 = F, 2 = M)			−0.020	(−0.240; 0.152)		−0.003	(−0.229; 0.215)		−0.037	(−0.309 0.124)
Age			−0.096^*^	(−0.172; −0.007)		−0.049	(−0.142; 0.046)		−0.021	(−0.113; 0.069)
Educational level			−0.120^*^	(−276; −0.032)		−0.007	(−0.129; 0.148)		−0.067	(−0.232; 0.037)
Financial status^1^			−0.020	(−0.077; 0.155)		0.038	(−0.071; 0.147)		0.057	(−0.044; 0.169)
Income level			−0.025	(−0.152; 0.092)		−0.033	(−0.179; 0.098)		−0.020	(−161; 0.108)
F		26.033	14.505		14.688	7.450		33.896	17.310	
R^2^		0.250	0.264		0.165	0.146		0.314	0.321	
Model *p*		<0.001	<0.001		<0.001	<0.001		<0.001	<0.001	

Standardized beta coefficients.

*** *p* ≤ 0.001;

** *p* ≤ 0.01;

* *p* ≤ 0.05. *N* = 377.

1 Self-reported.

Results also indicate that, after controlling for demographic variables, social power moderated perceptions of warmth (β = 0.096, t = 2.014, *p* < 0.045), competence (β = 0.252, t = 4.903, *p* < 0.001), and agency (β = 0.119, t = 2.573, *p* < 0.010).

With respect to perceptions of warmth, simple slope analyses showed that the simple slope was significant for high social power (one standard deviation above the mean; ß = −0.519, t = −8.530, *p* < 0.001) as well as for low social power (one standard deviation below the mean; ß = −0.417, t = −6.836, *p* < 0.001). However, the two slopes were not significantly different (t = 1.182, df = 750, *p* = 0.237), indicating that the moderating effect of social power on perceptions of warmth lacks statistical robustness and should be interpreted as non-significant.

**Figure 2:**
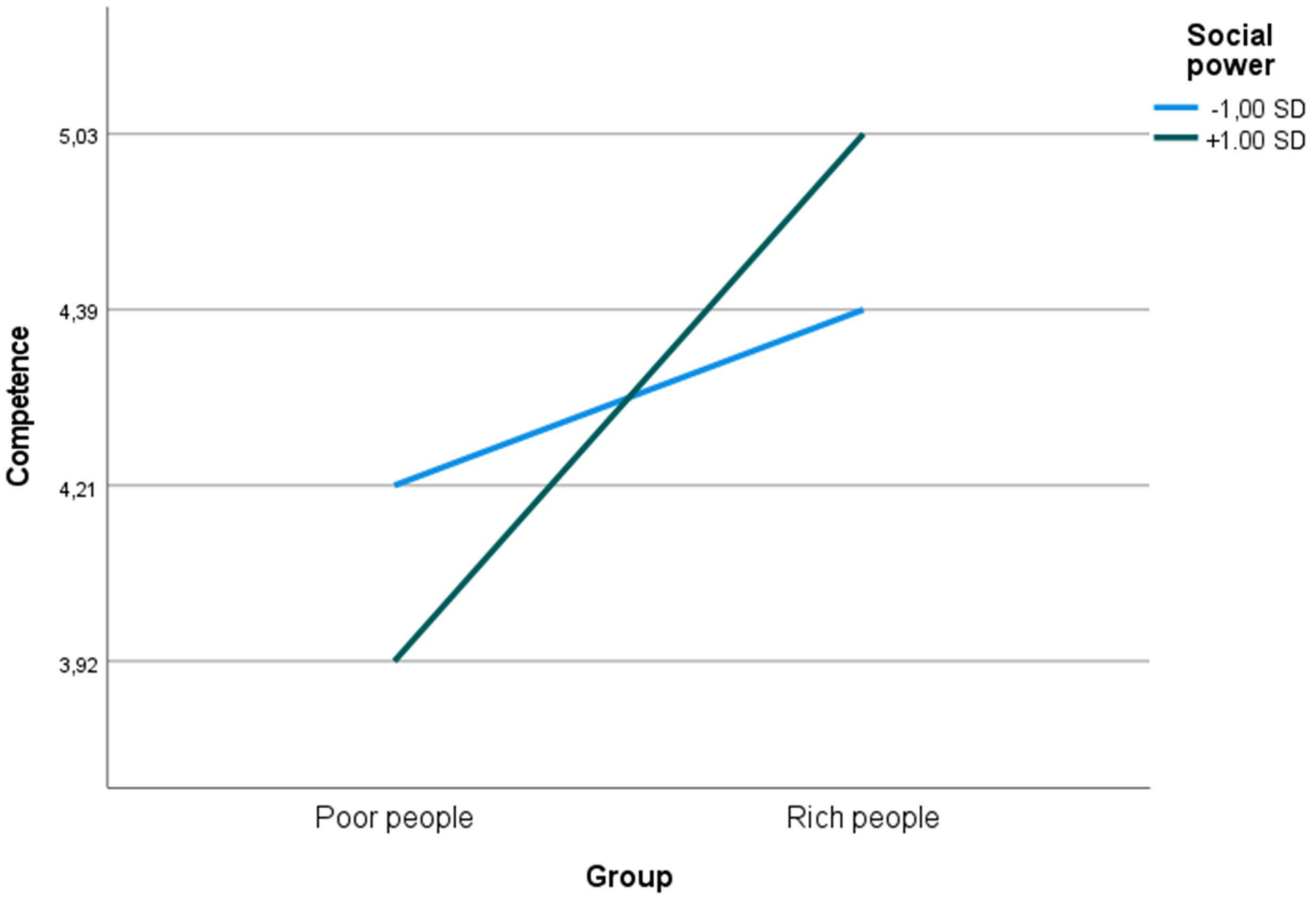
Predicted perceptions of competence at high and low levels of social power.

With respect to perceptions of competence, in contrast, simple slope analyses revealed that the simple slope was significant for low social power (one standard deviation below the mean; ß = −0.552, t = 8.036, *p* < 0.001), but not for high social power (one standard deviation above the mean; ß = 0.086, t = 1.257, *p* = 0.209), and that the two slopes were significantly different (t = 4.810, df = 750, *p* < 0.001), indicating that compared to high social power, low social power decreased the difference in perceptions of competence between the two groups (rich and poor; see Figure 2).

**Figure 3:**
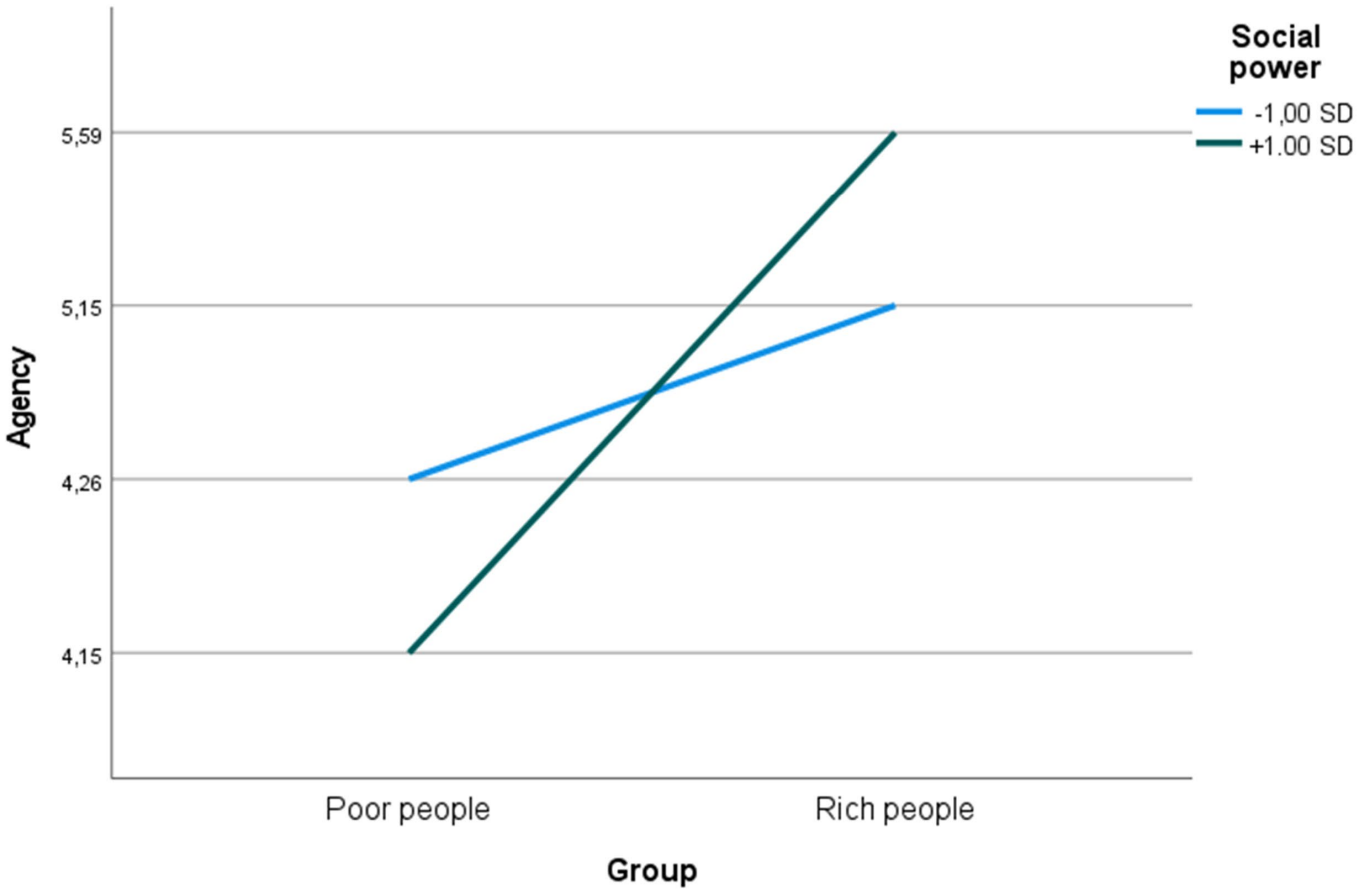
Predicted perceptions of agency at high and low levels of social power.

Finally, considering perceptions of agency, the simple slope was significant for high social power (ß = 0.442, t = 6.636, *p* = < 0.001) and low social power (ß = 0.721, t = 10.785, *p* = <0.001), and the two slopes were significantly different (t = 2.872, df = 750, *p* = 0.004), indicating that differences in perceptions of the rich vs. poor with respect to agency were larger at high compared to low social power (see Figure 3).

In sum, results indicate that the two types of power have different effects on perceptions of warmth and competence, as well as agency. Hence, results offer support to the hypothesis.

## Discussion

The findings from the present study demonstrate that social and personal power can have different effects on stereotyping involving the two universal dimensions of social perception: warmth and competence (e.g., Fiske et al., 2002, 2007; Cuddy et al., 2008), as well as agency, which was identified as a separate dimension in the present study.

Hence, the findings offer support to Lammers et al. (2009), who proposed that social and personal power can produce different effects on stereotyping. The findings reveal that personal power significantly moderated perceptions of warmth, but not perceptions of competence or agency, while social power primarily moderated perceptions of competence and agency. The results pertaining to competence and agency reveal a similar pattern, which is in line with previous research that conceptualizes competence and agency as closely related, yet not entirely overlapping (Cuddy et al., 2008). Hence, the findings from the present study indicate that social and personal power can produce a reversed pattern of effects on stereotyping involving warmth and competence.

Based on the pattern of results in the present study, however, it cannot be easily evaluated whether one type of power promoted more stereotyping compared to the other, i.e., if social power produced less stereotyping compared to personal power, as proposed by Lammers et al. (2009). Replications across different groups or individuals as stereotyping targets are needed to more reliably test this proposition.

When considering the results from the present study in view of the original study by Lammers et al. (2009), several differences that may have affected the results should be noted. While the present study investigated three dimensions of stereotyping (i.e., warmth, competence, and agency) of a group target, Lammers et al. (2009) investigated unidimensional stereotyping of an individual target, i.e., the degree to which a female was perceived as stereotypically female. Their sample was also considerably smaller (*N* = 113, divided into six experimental groups) and comprised university students at a considerably younger age compared to the present sample. Lammers et al. (2009) also used priming to manipulate social or personal power, whereas the present study measured participants’ self-reported social and personal power. As previously noted, Mayiwar and Lai (2019) replicated the experiment by Lammers et al. (2009) with a larger sample (*N* = 295), and the replication produced considerably weaker and partially inconclusive results (but see Lammers and Stoker, 2019). Hence, it is possible that the differences in results between the abovementioned studies and the present study may be ascribed to differences in sampling, stereotyping tasks, and methodological approach. For example, it is possible that stereotype content as well as the degree of stereotyping vary with age (cf. Woods et al., 2005).

When considering the findings from the present study in view of the stereotype content model (SCM) and the associated BIAS map (Cuddy et al., 2007, 2008), the pattern of findings indicates that high personal power, by means of elevated perceptions of warmth, is associated with more paternalistic stereotyping of the poor, i.e., stereotyping based on pity rather than contempt, while rich people are more enviously stereotyped, i.e., rated lower on warmth (see Figure 1).

With respect to social power, in contrast, the pattern of findings may suggest that high social power promotes more contemptful perceptions of poor people’s competence and agency, as well as more envious or admiring perceptions of rich people’s competence and agency (see Figures 2, 3). It is also possible that degree to which stereotyping of the rich is envious vs. admiring is influenced by the way in which power is conceptualized (cf. Scholl et al., 2020). For example, it seems plausible to speculate that viewing power as opportunity will lead to more envious stereotyping, whereas viewing power as responsibility will promote more admiring stereotyping. The distinction between conceptualizing social power as opportunity vs. responsibility and its effects on stereotyping should therefore be subject to further scrutiny.

The effects of social power (as opposed to personal power) on perceptions of competence and agency observed in the present study may also be considered in light of recent studies using alternative measures of power. For example, Yzerbyt et al. (2022) reason that judgments of competence provide opportunity to defend or to regain positive identity perceptions, depending on the observer’s position in the social hierarchy. Moreover, they argue that when economic resources, power, and status are undisputable characteristics of a target, which should be the case for the two groups investigated here, a high level of agreement can be expected in perceptions of competence and agency. Their findings also suggest that the overlap in social judgments of ability (competence) and assertiveness is higher among high-status observers compared to low-status observers, which in turn may represent a partial explanation for why social power, which is derived from social interaction rather than independence (personal power), was found to significantly influence perceptions of competence as well as agency, and in a similar direction, whereas personal power was not.

Another and related potential explanation for the pattern of findings pertaining to social power is the presence of an ingroup effect, i.e., that powerful people prefer to view powerful people (rich people) as competent and warm. However, Norway is characterized by a high level of income and economic equality, and poor people and rich people are generally considered out-groups. [In the current sample, 3.4% reported a very low (<NOK 300.000) personal income, whereas 1.9% reported what is generally considered a very high (>NOK 2 million) personal income.]

Yet, it may be argued that as part of the research design, powerful participants were asked to rate powerful (rich people) and powerless participants were asked to rate powerless (poor people). However, perceptions of competence (and agency) were affected by social power only, whereas personal power had no effect, and the differences in effects between personal and social power are of primary interest here. Future studies may, nevertheless, benefit from using other social groups, i.e., ingroups as well as outgroups, in which the power dimension is less salient.

It should also be noted that the sample was skewed in terms of gender distribution, with women comprising the majority (74.3%) of the sample. Previous research has shown that women are more likely to participate in voluntary, non-compensated online surveys than men (Keusch, 2015). The gender distribution was skewed in the same direction in the studies by Lammers et al. (2009) and Mayiwar and Lai (2019). However, no correlation between participants’ gender and any dependent or independent variables was identified in the present study. Yet, future studies may benefit from using samples with a more even gender distribution in order to test potential interaction effects involving gender.

Future studies are needed to test the generalizability of the pattern of findings presented here across settings, samples, and social groups or individuals. Future studies on the effects of social and personal power on stereotyping may also benefit from investigating the role of perceived status and competition and the distinct emotions associated with different patterns of stereotyping, e.g., pity, envy, admiration, and contempt, in order to shed light on potential moderators and other explanatory mechanisms (cf. Fiske et al., 2002; Cuddy et al., 2008).

## Conclusion

The present study contributes to existing research on the effects of power on stereotyping. The findings suggest that two types of power, i.e., social power and personal power, produce different effects on perceptions of warmth and competence, as well as of agency, within and across two groups: rich people and poor people. These findings highlight the need for more research exploring the malleability of power effects in general and the effects of social vs. personal power on social perception and stereotyping in particular.

## Data availability statement

The raw data supporting the conclusions of this article will be made available by the authors, without undue reservation.

## Ethics statement

The present study was designed in accordance with the European Data Protection Regulation (GDPR) and no sensitive personal data were collected. Data collection was performed using the digital survey tool Nettskjema, which is offered by the University of Oslo (Norway) and specifically designed to meet Norwegian privacy requirements. All potential participants were informed that (1) participation was voluntary and could be terminated at any time without providing any reason, and (2) the study was strictly anonymous and designed in accordance with privacy regulations, i.e., that no information that could identify individual participants directly or indirectly was collected. All participants were required to provide their informed consent.

## Author contributions

The author confirms being the sole contributor of this work and has approved it for publication.

## Conflict of interest

The author declares that the research was conducted in the absence of any commercial or financial relationships that could be construed as a potential conflict of interest.

## Publisher’s note

All claims expressed in this article are solely those of the authors and do not necessarily represent those of their affiliated organizations, or those of the publisher, the editors and the reviewers. Any product that may be evaluated in this article, or claim that may be made by its manufacturer, is not guaranteed or endorsed by the publisher.
